# Association between folate metabolism polymorphisms and breast cancer: a case-control study

**DOI:** 10.1590/1678-4685-GMB-2020-0485

**Published:** 2021-10-22

**Authors:** Ana Paula D’Alarme Gimenez-Martins, Márcia Maria Urbanin Castanhole-Nunes, Carlos Henrique Viesi do Nascimento-Filho, Stéphanie Piacenti dos Santos, Ana Lívia Silva Galbiatti-Dias, Glaucia Maria de Mendonça Fernandes, Caroline Izak Cuzziol, José Luis Esteves Francisco, Érika Cristina Pavarino, Eny Maria Goloni-Bertollo

**Affiliations:** 1 Faculdade de Medicina de São José do Rio Preto (FAMERP), Departamento de Biologia Molecular, Unidade de Pesquisa em Genética e Biologia Molecular (UPGEM), São José do Rio Preto, SP, Brazil.; 2 Faculdade de Medicina de São José do Rio Preto (FAMERP), Departamento de Ginecologia e Obstetrícia, São José do Rio Preto, SP, Brazil.; 3 Fundação da Faculdade Medicina de São José do Rio Preto (FUNFARME), São José do Rio Preto, SP, Brazil.

**Keywords:** Breast carcinoma, genetic polymorphism, methylenetetrahydrofolate reductase (*MTHFR*), methionine synthetase (*MTR*), methionine synthase reductase (*MTRR*)

## Abstract

We investigated the association between methylenetetrahydrofolate reductase (*MTHFR* C677T and A1298C), methionine synthetase (*MTR* A2756G), and methionine synthase reductase (*MTRR* A66G) polymorphisms involved in folate pathway and breast cancer risk, and the interaction between these polymorphisms and tobacco and alcohol consumption. Furthermore, we evaluated the association between these polymorphisms and clinicopathological variables. This case-control study included 606 Brazilian women, comprising 128 patients with breast cancer and 478 controls. *MTHFR* and MTR polymorphisms were genotyped using polymerase chain reaction-restriction fragment length polymorphism (PCR-RFLP) and *MTRR* polymorphisms using real-time PCR. Age ≥50 years (odds ratio [OR]: 2.65; 95% confidence interval [CI]: 1.65-4.26; *p*<0.001) and alcohol consumption (OR: 1.76; 95% CI: 1.0-2.85; *p*=0.021) were associated with an increased risk of breast cancer. For *MTHFR* A1298C, we observed a reduced risk of developing breast cancer in the codominant model (genotype CC-OR: 0.22; 95% CI: 0.06-0.74; *p*=0.014), recessive model (OR: 0.22; 95% CI: 0.07-0.76 *p*=0.004), and log-additive model (OR: 0.70; 95% CI: 0.49-0.98; *p*=0.035). Women aged ≥50 years and those who are alcohol consumers had increased susceptibility to breast cancer, and *MTHFR* A1298C modulated the risk for this disease. This is the first study to evaluate the association between polymorphisms in folate metabolism and breast cancer in the northwest region of São Paulo State, Brazil.

## Introduction

Breast cancer is the most common cancer and the second most common cause of mortality (INCA, 2019; Siegel et al., 2021). Thus, the discovery of predictors of breast cancer plays a central role in monitoring women and early detection, which increases the chances of curative treatment. Factors related to developed countries linked to increased breast cancer cases include early menarche, older age upon first occasion of giving birth, nulliparity, obesity, alcohol consumption, breastfeeding, and physical activity (Key et al., 2001; Arthur et al. 2020). Moreover, the identification of genetic markers related to breast cancer development is fundamental for guiding therapeutic approaches (Lee et al., 2017). Using genetic risk profiles, personalized medicine can be achieved (Naushad et al., 2012). 

There are potential biomarkers, such as polymorphisms of the folate pathway or one-carbon pathway. The latter pathway is responsible for the donation of the methyl group, which is further used in important processes such as DNA methylation, histone methylation, and protein methylation (Mentch and Locasale, 2016; Hiraoka and Kagawa, 2017). Folate acid and diet provide the methyl group, leading to the conversion of dihydrofolate to tetrahydrofolate. Subsequently, tetrahydrofolate is converted to 5,10-methylenetetrahydrofolate, which donates a methyl group for DNA synthesis and converges to 5-methyltetrahydrofolate, a reaction catalyzed by the methylenetetrahydrofolate reductase (MTHFR) enzyme (Hiraoka and Kagawa, 2017; Scaglione and Panzavolta, 2014). Under methionine synthetase (MTR) and methionine synthase reductase (MTRR) enzyme activity, 5-methyltetrahydrofolate is converted to tetrahydrofolate, and the donated methyl group is used in methylation circuits, including the conversion of homocysteine to methionine, and is pivotal to the activity of S-adenosyl-L-methionine (SAM) synthase and DNA methyltransferases (Galbiatti, et al., 2012a; Mentch and Locasale, 2016; Hiraoka and Kagawa, 2017). 

The function of this pathway is not only restricted to methylation events but also to DNA synthesis, thereby playing an important role in cancer development. Therefore, to better understand the activity of these enzymes, it is necessary to understand the effects of single nucleotide polymorphisms (SNPs) on cancer susceptibility. The *MTHFR* C677T polymorphism has been associated with susceptibility to developing breast cancer among Asian women, but no association was observed in Caucasians (Chen et al., 2019), suggesting the relevance of ethnicity and region. Interestingly, SNPs in the one-carbon pathway were differentially methylated in breast tissues from healthy women (Song et al., 2016), indicating the role of these SNPs in methylation circuits. Taken together, these findings remain ambiguous, and more case-control studies are required.

Here, we aimed to investigate the association between *MTHFR* C677T (rs1801133), *MTHFR* A1298C (rs1801131), *MTR* A2756G (rs1805087), and *MTRR* A66G (rs1801394) polymorphisms and the risk of developing breast cancer in women from Northwest São Paulo State, Brazil. We further examined the association of risk factors, including age, smoking habits, alcohol consumption, number of pregnancies, body mass index, hormone therapy, and clinicopathological variables, with breast cancer development. Overall, we showed that the *MTHFR* A1298C polymorphism reduced the risk of developing breast cancer, whereas alcohol intake increased the risk.

## Subjects and Methods

### Study population

The present case-control study was approved by the National Ethics Committee (CAAE - 04069612.1.0000.5415), Sao Jose do Rio Preto Medical School (FAMERP), Sao Paulo, Brazil (process number: 84397). All individuals agreed with the informed consent form, according to Resolution 466/12 of the National Health Council (CONEP). This study is consistent with STrengthening the Reporting of Observational Studies in Epidemiology (STROBE) (Gallo et al., 2011).

We evaluated 606 eligible women, comprising 128 patients (case group) after total excision or biopsy were diagnosed and histologically confirmed to have breast cancer and 478 healthy women volunteers (control group); only women without any history of cancer or familial history of cancer aged >40 years were included. All women were admitted between January 2013 and January 2015 to the Hospital de Base, São Jose do Rio Preto Medical School, São José do Rio Preto, Brazil. 

The control group was recruited from blood donors who met the government guidelines for blood donor selection (http://www.hemonline.com.br/portarias/rdc153/indexframe.htm). During recruitment of the control group, efforts were made to maintain a similarity in age and geographic region between the control and case groups. In individuals who were most recently recruited to the control group (n=219), we also performed a standardized questionnaire to obtain personal data, demographic and epidemiological factors such as lifestyle (smoker and alcohol drinker), reproductive history (number of live births, number of miscarriages, number of stillbirths, and use of contraceptives), and family history of cancer (breast cancer and/or other cancers). Measurements of height and weight were used to calculate the body mass index (BMI). 

Additional information about the diagnosis, histological type, tumor location, tumor size (T), regional lymph node involvement (N), presence of metastasis (M), progesterone receptor (PR), estrogen receptor (ER), and human epidermal growth factor receptor-type 2 (HER2) overexpression were obtained from the medical records and pathology reports. TNM classification of malignant tumors was classified according to the UICC/AJCC of 2010 (Sobin et al., 2010) and grouped into stage I (T1N0), stage II (T1N1 and T2N0-1), stage III (T3N0-1 and T1-3N2), and stage IV (T4N0-3, T1-3N3, and T1-4N0-3M1) according to the AJCC Cancer Staging Manual (Fleming et al., 1997). Tumor stages I and II were considered non-advanced, while stages III and IV were considered advanced. The clinicopathological characteristics of intrinsic breast cancer subtypes were defined according to Perou et al. (2000) and Goldhirsch et al. (2011): Luminal A: ER- and/or PgR-positive, HER2 negative and Ki-67 <14%; Luminal B (HER2-negative): ER- and/or PgR-positive, HER2-negative and Ki-67 high; Luminal B (HER2-positive): ER- and/or PgR-positive, any Ki-67, HER 2 overexpressed or amplified; HER2-positive: ER and PgR absent and HER2 overexpressed or amplified; and “basal-like” (triple-negative): ER and PgR absent and HER2-negative (Perou *et al.*, 2000; Goldhirsch *et al*., 2011)

We considered smokers who consumed at least 100 cigarettes during their lifetime and alcohol drinkers as those who consumed more than one drink weekly (one drink corresponding to approximately 44 mL of liquor, or 118 mL of wine, or 350 mL of beer) (Carpenter and Cohen, 1990).

### Genotyping

Genomic DNA was extracted from peripheral blood leukocytes according to a previously detailed method (Miller et al., 1988) with modifications. *MTHFR* C677T (rs1801133), *MTHFR* A1298C (rs1801131), and *MTR* A2756G (rs1805087) polymorphisms were determined using polymerase chain reaction-restriction fragment length polymorphism (PCR-RFLP) with the following primers: *MTHFR* C677T, sense 5′-TGA AGG AGA AGG TGT CTG CGG GA-3′, anti-sense 5′-AGG ACG GTG CGG TGA GAG TG-3′; and *MTR* A2756G, sense 5′-CCAGGG TGC CAG GTA TAC AG-3′, anti-sense 5′-GCC TTT TAC ACT CCT CAA AAC-3′. Genotyping of the *MTHFR* C677T polymorphism was accomplished using the restriction enzyme, HinfI. The resulting fragments were 198 bp (C allele), 175 bp, and 23 bp (T allele). The *MTR* A2756G polymorphism was genotyped using the restriction enzyme, *Hae*III. The resulting fragments were 413 and 85 bp (A allele) and 290, 123, and 85 bp (G allele) (Galbiatti et al., 2010; Barbosa et al., 2012; Zara-Lopes et al., 2016). Genotyping of *MTRR* A66G (rs1801394) polymorphisms was performed using the Real Time PCR-SNP Genotyping Assay (Thermo Fisher Scientific, Carlsbad, CA) *-* Assay ID: C_3068176_10 (20X), in following the manufacturer’s instructions. The reactions were performed using the StepOnePlus™ Real-Time PCR System (Galbiatti *et al*., 2012b) (Table 1). To improve the reliability of the analysis, we performed genotyping confirmation in 10% of cases and control samples randomly for each polymorphism. We found 100% concordance.

**Table 1 ‒:** Primers sequence, restriction enzyme and fragments size.

Polymorphisms	Primers Sequence	Restriction Enzyme	Fragments Size
*MTHFR C677T*		*Hinf* I	
sense	5’- TGA AGG AGA AGG TGT CTG CGG GA 3’		C allele - 198 bp
anti-sense	5’- AGG ACG GTG CGG TGA GAG TG 3’		T allele - 175, 23 bp
*MTHFR A1298C*		*Mbo* II	
sense	5’- CAA GGA GGA GCT GCT GAA GA 3’		A allele - 72, 28 bp
anti-sense	5’ - CAA CTC CAG CAT CAC TCA CT 3’		C allele - 100, 28 bp
*MTR A2756G*		*Hae* III	
sense	5’- CCA GGG TGC CAG GTA TAC AG 3’		A allele - 413, 85 bp
anti-sense	5’- GCC TTT TAC ACT CCT CAA AAC 3’		G allele-290, 123, 85 bp

MTHFR - Methylenetetraydrofolate reductase; MTR - methionine synthase, bp- base-pair‘

### Statistical analysis

Multiple logistic regression was used to determine the effects of variables on breast cancer risk and clinicopathological characteristics using the Minitab/Windows program - Version 14.0. The model included the following variables: age (reference: <50 years old; median), smoking habits (reference: non-smoker), alcohol consumption (reference: no alcohol drinkers), number of pregnancies (reference: ≥3 pregnancies, BMI (reference: <25 kg/m²), hormone therapy (reference: not using hormone therapy), *MTHFR* C677T (reference: genotype CC), *MTHFR* A1298C (reference: genotype AA), *MTR* A2756G (reference: genotype AA), and *MTRR* A66G (reference: genotype AA). The clinicohistopathological characteristics were T classification (reference: T1 and T2), N classification (reference: no lymph nodes affected), and M classification (reference: no metastasis). Logistic regression was adjusted for age, sex, smoking habits, and alcohol consumption. 

The Hardy-Weinberg equilibrium (HWE) was assessed using the chi-square test using the program SNPStats (available at http://bioinfo.iconcologia.net/SNPstats_web). The SNPstats online computer program was used to analyze the following models: 1) codominant (heterozygous vs. homozygous wild type and polymorphic homozygous vs. homozygous wild type), 2) dominant (heterozygous plus polymorphic homozygous vs. homozygous wild type), 3) recessive (polymorphic homozygous vs. homozygous wild type plus heterozygous), 4) overdominant (wild homozygous vs. heterozygous plus polymorphic homozygous), and 5) additive (weight polymorphic homozygote vs. heterozygote 2 plus homozygous wild-type) (Fernandes et al., 2016). The relationship between polymorphisms and clinicohistopathological features was also analyzed using SNPStats. All results are presented as odds ratios (OR) with a 95% confidence interval (95% CI); p-values <0.05 were considered statistically significant.

## Results

In this study, we observed that women aged ≥50 years old (OR: 2.65; 95% CI: 1.65-4.26; *p*<0.001) and who consumed alcohol (OR: 1.76; 95% CI: 1.09-2.85; *p*=0.021) had an increased risk of breast cancer. Smoking habits, number of pregnancies, BMI ≥25 kg/m^2^, and hormone therapy showed no association with the risk of breast cancer (data not shown). Regarding clinicohistopathological characteristics, we found that 94.6% of all breast cancers were invasive ductal carcinomas, 4.6% had lobular carcinoma, and 0.8% mixed ductal and lobular carcinoma. Regarding TNM classification, 62.5% of cases were T1 or T2, 42.2% of cases had affected lymph nodes, and 34.4% of cases had metastasis. The frequency of breast cancer subtypes was as follows: 48.4% for luminal B, 26.6% for luminal A, 12.5% for triple-negative, and 10.1% for HER2 overexpression.

The analyses of *MTHFR* C677T (rs1801133), *MTHFR* A1298C (rs1801131), *MTR* A2756G (rs1805087), and *MTRR* A66G (rs1801394) polymorphisms; tumor size; lymph node involvement; and metastasis are shown in Table 2. We did not find a significant association between polymorphisms and clinical features. Regarding SNPs and breast cancer subtypes, we did not observe an association between luminal A, luminal B, HER2 overexpression, and triple-negative subtypes and polymorphisms (Table 3). We observed that the *MTHFR* A1298C (rs1801131) polymorphism reduced the risk of developing breast cancer in the codominant model (genotype CC - OR: 0.22; 95% CI: 0.06-0.74; *p*=0.014), recessive model (OR: 0.22; 95% CI: 0.07-0.76; *p*=0.004), and log-additive model (OR: 0.70; 95% CI: 0.49-0.98; *p*=0.035). No significant association was found between the *MTR* A2756G (rs1805087), *MTRR* A66G (rs1801394), and *MTHFR* C677T (rs1801133) polymorphisms analyzed and models (p>0.05). The genotype distributions of *MTHFR* C677T (rs1801133), *MTHFR* A1298C (rs1801131), *MTR* A2756G (rs1805087) and *MTRR* A66G (rs1801394) polymorphisms and breast cancer risk are presented in Table 4 (128 cases and 478 controls). 

**Table 2 ‒:** Association between *MTHFR* C677T, *MTHFR* A1298C, *MTR* A2756G and *MTRR* A66G polymorphisms and clinical tumor size (T1-T2 vs T3-T4), lymph nodes involvement and metastasis.

Clinical features	MTHFR C677T		MTHFR A1298C		MTR A2756G		MTRR A66G	
	CC	CT+TT	AA	AC+CC	AA	AG+GG	AA	AG+GG
	N (%)	N (%)	N (%)	N (%)	N (%)	N (%)	N (%)	N (%)
Tumor size								
T1-T2	29 (22.7)	51 (39.8)	49 (38.2)	31 (24.2)	50 (39.1)	30 (23.3)	25 (19.5)	55 (43.0)
T3-T4	16 (12.5)	31 (24.2)	29 (22.7)	18 (14.1)	32 (25.0)	15 (11.8)	22 (17.2)	25 (19.5)
Unknown	01 (0.8)	0 (0)	01 (0.8)	0 (0)	01 (0.8)	0 (0)	01 (0.8)	0 (0)
OR (95% CI)	1.00	1.31 (0.57-3.03)	1.00	1.18 (0.53-2.64)	1.00	0.77 (0.34-1.74)	1.00	0.50 (0.23-1.09)
*p value*	0.522		0.682		0.526		0.081	
Lymph nodes								
No	27 (21.1)	43 (33.6)	41 (32.0)	29 (22.7)	44 (34.4)	26 (20.3)	25 (19.5)	45 (35.2)
Yes	17 (13.2)	37 (28.9)	36 (28.0)	18 (14.1)	37 (28.9)	17 (13.2)	20 (15.6)	34 (26.5)
Unknown	02 (1.6)	02 (1.6)	02 (1.6)	02 (1.6)	02 (1.6)	02 (1.6)	03 (2.4)	01 (0.8)
OR (95% CI)	1.00	1.98 (0.83-4.71)	1.00	0.80 (0.35-1.81)	1.00	0.62 (0.27-1.43)	1.00	0.87 (0.39-1.95)
*p value*	0.122		0.591		0.264		0.730	
Metastasis								
No	29 (22.7)	51 (39.8)	44 (34.4)	36 (28.0)	48 (37.5)	32 (25.0)	30 (23.3)	50 (39.1)
Yes	16 (12.5)	28 (21.8)	32 (25.0)	12 (9.4)	31 (24.2)	13 (10.1)	15 (11.7)	29 (22.7)
Unknown	01 (0.8)	03 (2.4)	03 (2.4)	01 (0.8)	04 (3.2)	0 (0)	03 (2.4)	01 (0.8)
OR (95% CI)	1.00	1.19 (0.50-2.82)	1.00	0.48 (0.21-1.11)	1.00	0.51 (0.22-1.20)	1.00	1.06 (0.47-2.40)
*p value*	0.697		0.08		0.123		0.894	

OR, odds Ratio; Adjusted for age, smoking habits, alcohol consumption, gestations, BMI (Body-mass index), hormone therapy and polymorphisms.

**Table 3 ‒:** Association between *MTHFR* C677T, *MTHFR* A1298C, *MTR* A2756G and *MTRR* A66G polymorphisms and breast cancer subtype.

Breast Cancer Subtype	MTHFR C677T		MTHFR A1298C		MTR A2756G		MTRR A66G	
	CC	CT+TT	AA	AC+CC	AA	AG+GG	AA	AG+GG
	N (%)	N (%)	N (%)	N (%)	N (%)	N (%)	N (%)	N (%)
Luminal A	12 (9.4)	22 (17.2)	19 (14.8)	15 (11.7)	22 (17.2)	12 (9.4)	15 (11.7)	19 (14.8)
OR (95% CI)	1.00	1.01 (0.42-2.45)	1.00	1.64 (0.70-3.85)	1.00	1.07 (0.44-2.58)	1.00	0.62 (0.27-1.45)
*p value*	0.975		0.253		0.879		0.272	
Luminal B	22 (17.2)	40 (31.2)	39 (30.5)	23 (17.9)	38 (29.7)	24 (18.7)	18 (14.1)	44 (34.4)
OR (95% CI)	1.00	0.77 (0.34-1.71	1.00	0.91 (0.42-1.98)	1.00	1.39 (0.63-3.04)	1.00	2.16 (0.98-4.76)
*p value*	0.514		0.817		0.415		0.056	
HER2 Overexpression	04 (3.2)	09 (7.0)	08 (6.2)	05 (3.9)	10 (7.8)	03 (2.4)	06 (4.6)	07 (5.5)
OR (95% CI)	1.00	1.31 (0.33-5.29)	1.00	1.04 (0.29-3.73)	1.00	0.35 (0.08-1.50)	1.00	0.58 (0.16-2.08)
*p value*	0.704		0.948		0.156		0.405	
Triple Negative	05 (3.9)	11 (8.5)	12 (9.4)	04 (3.2)	10 (7.8)	06 (4.6)	07 (5.5)	09 (7.0)
OR (95% CI)	1.00	1.36 (0.40-4.57)	1.00	0.50 (0.14-1.75)	1.00	0.94 (0.29-3.09)	1.00	0.63 (0.20-1.95)
*p value*	0.622		0.276		0.923		0.425	
Unknown	03 (2.4)	0 (0)	01 (0.8)	02 (1.6)	03 (2.4)	0 (0)	02 (1.6)	01 (0.8)

OR, odds Ratio; Adjusted for age, smoking habits, alcohol consumption, gestations, BMI (Body-mass index), hormone therapy and polymorphisms.

**Table 4 ‒:** Analyzis of MTHFR C677T, MTHFR A1298C, MTR A2756G and MTRR A66G polymorphisms with breast cancer.

Model	Genotype	Case N (%)	Control N (%)	OR (95%CI)	p	Genotype	Case N (%)	Control N (%)	OR (95%CI)	*p*
	*MTHFR C677T*					*MTHFR* A1298C				
Codominant	CC	46 (35.9)	207 (43.3)	1.00 1.21 (0.77-1.89) 1.71 (0.91-3.22)	0.25	AA	79 (61.7)	261 (54.6)	1.00 0.90 (0.59-1.39) *0.22 (0.06-0.74)*	0.01^*^
	CT	61 (47.7)	218 (45.6)			AC	46 (35.9)	170 (35.6)		
	TT	21 (16.4)	53 (11.1)			CC	3 (2.3)	47 (9.8)		
	C allele	153 (60)	632 (66)			A allele	204 (80)	692 (72)		
	T allele	103 (40)	324 (34)			C allele	52 (20)	264 (28)		
	HWE	p=1.0	p=0.76			HWE	p=0.28	p=0.016		
Dominant	CC	46 (35.9)	207 (43.3)	1.00	0.21	AA	79 (61.7)	261 (54.6)	1.00	0.20
	CT+TT	82 (64.1)	271 (56.7)	1.31 (0.86-2.00)		AC+CC	49 (38.3)	217 (45.4)	0.76 (0.50-1.16)	
Recessive	CC+CT	107 (83.6)	425 (88.9)	1.00	0.15	AA+AC CC	125 (97.7)	431 (90.2)	1.00	0.004^*^
	TT	53 (11.1)	53 (11.1)	1.55 (0.87-2.75)		CC	3 (2.3)	47 (9.8)	0.22 (0.07-0.76)	
Overdominant	CC+TT	67 (52.3)	260 (54.4)	1.00	0.82	AA+CC	82 (64.1)	308 (64.4)	1.00	0.92
	CT	61 (47.7)	218 (45.6)	1.05 (0.70-1.58)		AC	46 (35.9)	170 (35.6)	1.02 (0.67-1.57)	
Log-additive	-	-	-	1.28 (0.95-1.74)	0.1	-	-	-	*0.70 (0.49-0.98)*	0.03^*^
	*MTR* A2756G					*MTRR* A66G				
Codominant	AA	83 (64.8)	300 (62.8)	1.00 1.22 (0.79-1.89) 0.23 (0.03-1.77)	0.12	AA	48 (37.5)	168 (35.1)	1.00 0.99 (0.63-1.56) 0.76 (0.42-1.35)	0.59
	AG	44 (34.4)	156 (32.6)			AG	57 (44.5)	211 (44.1)		
	GG	1 (0.8)	22 (4.6)			GG	23 (18.0)	99 (20.7)		
	A allele	210 (82)	756 (79)			A allele	153 (60)	547 (57)		
	G allele	46 (18)	200 (21)			G allele	103 (40)	409 (43)		
	HWE	p=0.07	p=0.78			HWE	p=0.46	p=0.03		
Dominant	AA	83 (64.8)	300 (62.8)	1.00	0.63	AA	48 (37.5)	168 (35.1)	1.00	0.66
	AG+GG	45 (35.2)	178 (37.2)	1.11 (0.721.71)		AG+GG	80 (62.5)	310 (64.8)	0.91 (0.60-1.39)	
Recessive	AA+AG	127 (99.2)	456 (95.4)	1.00	0.06	AA+AG	105 (82)	379 (79.3)	1.00	0.30
	GG	1 (0.8)	22 (4.6)	0.21 (0.03-1.64)		GG	23 (18)	99 (20.7)	0.76 (0.45-1.29)	
Overdominant	AA+GG	84 (65.6)	322 (67.4)	1.00	0.27	AA+GG	71 (55.5)	267 (55.9)	1.00	0.68
	AG	44 (34.4)	156 (32.6)	1.28 (0.83-1.98)		AG	57 (44.5)	211 (44.1)	1.09 (0.72-1.65)	
Log-additive	-	-	-	0.98 (0.67-1.43)	0.91	-	-	-	0.89 (0.67-1.17	0.40

^*^ *p* - values significant. OR, odds Ratio; Adjusted for age, smoking habits, alcohol consumption, gestations, BMI (Body-mass index), hormone therapy and polymorphisms.

HWE analysis (Table 4) showed that the case and control groups were in equilibrium for *MTHFR* C677T (rs1801133) (case, *p*=1.00; control, *p=*0.76) and *MTR* A2756G (rs1805087) (case, *p*=0.07; control, *p*=0.7) polymorphisms. In the control group, *MTHFR* A1298C (rs1801131) and *MTRR* A66G (rs1801394) polymorphisms showed disequilibrium (*p*=0.01 and *p*=0.03). The frequencies of variant alleles for SNPs (Table 4) were: allele C, 60% case and 66% control for the *MTHFR* C677T (rs1801133) polymorphism; allele A, 80% case and 72% control for the *MTHFR* A1298C (rs1801131) polymorphism; allele A, 82% case and 79% control for the *MTR* A2756G (rs1805087) polymorphism; and allele A, 60% case and 57% control for the *MTRR* A66G (rs1801394) polymorphism.

## Discussion

Our results showed that age ≥50 years (*p*<0.001) and alcohol consumption (*p*=0.021) were risk factors for breast cancer in women. Women aged ≥50 years have been associated with an increased risk of breast cancer in other studies (Sangrajrang et al., 2010; Akilzhanova et al., 2013; He et al., 2014; Jiang-Hua et al., 2014; Gong et al., 2015). Age is also associated with menopause, body-mass index, and hormone therapy due to the influence of hormones during women’s lives (Hvidtfeldt et al., 2015). In a pooled cohort study, these authors. found that overweight women (median age, 56 ± 5.0 years) under hormone therapy had an increase in the absolute risk of breast cancer (p interaction=0.003) (Hvidtfeldt *et al*., 2015). Also, the risk of breast cancer increases with the levels of endogenous estradiol (Key et al., 2002). In their case-control study (313 cases and 626 controls), Ericson and colleagues confirmed the association between menopausal hormone therapy and breast cancer risk (*p*<0.001) in women in Malmo (Ericson et al., 2009). The present study did not evaluate the effects of menopausal hormone therapy. However, we did evaluate hormone use during women’s life, and found no statistically significant relationship between hormone use and breast cancer risk, as well as between BMI ≥ 25 kg/m^2^, smoking habits, and number of pregnancies, and breast cancer risk/development. In contrast, we observed an association between alcohol consumption and breast cancer risk. Consistent with our findings, Ma *et al*. (2009) and Ericson *et al*. (2009) also found a statistically significant association between alcohol intake and breast cancer. Alcohol could be an important factor for inducing carcinogenesis because it may induce epigenetic alterations, aberrant DNA methylation, and reduced folate absorption (Varela-Rey et al., 2013).

Regarding polymorphisms, we found disequilibrium in the control group for *MTHFR* A1298C (rs1801131) and *MTRR* A66G (rs1801394) polymorphisms. HWE represents the non-evolution and homogeneity of a population. However, the diversity of this population, models, and complexity of the diseases can lead to an imbalance between sample groups; therefore, a control group is more likely to be in disequilibrium (Wittke-Thompson et al., 2005).

In the present study, we did not find a significant association between *MTHFR* C677T, *MTR* A2756G, and *MTRR* A66G polymorphisms and breast cancer risk. Surprisingly, the *MTHFR* A1298C polymorphism showed a protective effect on breast cancer development, which opens the door for new investigations. The role of this polymorphism in cancer susceptibility is controversial, especially when considering patient ethnicity (Huang et al., 2007; Li et al., 2014). From this perspective, the current investigation in a Latino population is an important piece of the puzzle. From a biochemical perspective, the *MTHFR* A1298C polymorphism shows decreased enzymatic activity, although not as low as that of the C677T polymorphism and, therefore, might dysregulate the balance of DNA synthesis/methylation mechanisms (Weisberg et al., 1998). Altogether, the role of these polymorphisms in cancer development is uncertain, which points toward additional effects to be uncovered. Moreover, the balance of DNA synthesis/methylation circuits might be more complex than anticipated (Huang *et al*., 2007; Li *et al*., 2014).

Studies of C677T and A1298C polymorphisms in the *MTHFR* gene have shown that these SNPs decrease enzyme activity and alter folate levels, which leads to carcinogenesis (Suzuki et al., 2008; Ericson et al., 2009; Carvalho *et al*., 2012; Barbosa et al., 2012; Babyshkina et al., 2013; Jiang-Hua et al., 2014). A meta-analysis by Li *et al*., involving 57 case-control studies, suggested that C677T polymorphisms in the *MTHFR* gene may contribute to breast cancer development, while the A1298C polymorphism was not associated with disease risk (Li *et al*., 2014). However, another study in Thai women found no association between C677T and A1298C, partially corroborating our findings (Sangrajrang et al., 2010). Sharp and colleagues reported that the *MTHFR* A1298C SNP in a case-control study (62 women and 64 controls) reduced breast cancer risk in women with the 1298CC genotype compared to that of the AA genotype in the Scotland population (Sharp *et al*., 2002). Moreover, a study with Kazakhstan women found a 1.2-fold decrease in breast cancer risk in a dominant model (Akilzhanova et al., 2013). These findings are consistent with our results in which we observed that *MTHFR* A1298C polymorphism reduced breast cancer risk in a codominant model, recessive model, and log-additive model.

In this study, we did not observe an association between *MTR* A2756G and *MTRR* A66G polymorphisms and breast cancer development. A meta-analysis involving 12 case-control studies for *MTR* A2756G SNP, seven studies for *MTRR* A66G polymorphisms (Weiner et al., 2012), and two studies involving Chinese women also found no association between *MTR* A2756G polymorphism and breast cancer (He et al., 2014; Jiang-Hua et al., 2014). Consistent with our findings, other studies, and the study by Naushad et al., which included 244 case-control pairs of Indian women (Naushad et al., 2012) also found no significant association between the *MTRR* A66G polymorphism and breast cancer risk (Kotsopoulos et al., 2008; Sangrajrang et al., 2010). 

Regarding breast cancer subtype, our study observed no association between the polymorphisms studied and luminal A, luminal B, HER2 overexpression, and triple-negative subtypes. Naushad et al. (2012) showed that the *MTHFR* C677T polymorphism is associated with a risk for the luminal B subtype, and the *MTR* A2756G polymorphism was associated with an increased risk for the luminal A subtype. For the *MTRR* A66G polymorphism, they also did not observe an association between any breast cancer subtypes, as in this study (Naushad *et al*., 2012). Batschauer et al. (2011) found no association between the *MTHFR* C677T polymorphism and HER2 receptor or ER and PR. Babyshkina et al. (2013) found that patients with at least one polymorphic allele for the *MTHFR* C677T polymorphism were significantly associated with a positive ER. For *MTHFR* A1298C, *MTR* A2756G, and *MTRR* A66G polymorphisms, Babyshkina *et al*. (2013) did not observe an association with breast cancer subtype. Tumors negative for hormone receptors and positive for HER2 have a worse prognosis in breast cancer (Lang et al., 2009; Batschauer *et al*., 2011); however, they may not be associated with *MTHFR* C677T, *MTHFR* A1298C, *MTR* A2756G, and *MTRR* A66G polymorphisms in folate metabolism. 

Our study is the first to show the profile of the female population from Northwestern São Paulo State, Brazil. Since few studies have been performed analyzing folate metabolism polymorphisms and clinical and histopathological features of breast cancer. Overall, this study states that age ≥50 years and alcohol consumption increases the risk of breast cancer in women. We did not find a significant association between *MTHFR* C677T, *MTR* A2756G, and *MTRR* A66G polymorphisms and breast cancer development. However, women with the 1298CC polymorphic genotype of the *MTHFR* gene had a lower risk of breast cancer development. Therefore, further studies are warranted to confirm the role of these SNPs in breast cancer development.
